# Propagation rules of shock waves in confined space under different initial pressure environments

**DOI:** 10.1038/s41598-022-18567-0

**Published:** 2022-08-23

**Authors:** F. Q. Wang, Q. Wang, Y. J. Wang, Z. M. Li, R. Li, X. C. Li, L. A. Yang, J. W. Lu

**Affiliations:** 1 School of Chemical Engineering, Anhui University of Science & Technology, Huainan, 232001 China; 2 Engineering Laboratory of Explosive Materials and Technology of Anhui Province, Huainan, 232001 China; 3 BGRIMM Technology Group, Beijing, 100160 China; 4 School of Civil Engineering and Architecture, Anhui University of Science & Technology, Huainan, 232001 China

**Keywords:** Energy science and technology, Engineering

## Abstract

In this paper, an initial pressure adjustable explosion vessel was developed, and the effect of negative pressure, positive pressure (0.2–1.8 atm) different initial ambient pressure on the explosive shock wave generated by the explosion of explosives was studied. The relationships between the specific impulse, shock wave velocity, the amount of explosive gas products and the ambient pressure were analyzed for different initial pressure environments. It was found that: the overpressure of the blast shock wave decreases with the initial ambient pressure of the explosion, and there exists a negative pressure environment with a dramatic pressure decrease near 0.6 atm, defined as the super-sensitive negative pressure P_cr_. The propagation velocity of an explosive wave increases with a decrease in the ambient pressure, and the propagation velocity at a pressure of 1.8 atm is four times less than the velocity at a pressure of 0.2 atm. The production of explosive gas products did not change. The greater the initial pressure of the environment where the explosive is located, the smaller the ratio of the gas generated by the explosion to the initial force gas in the explosion vessel is, and the greater the impact on the propagation of shock waves is. The maximum attenuation of the first specific impulse *i*_1_ is 72.97% and the maximum attenuation of the second specific impulse *i*_2_ is 72.39%. The experiments provide reference data for high-altitude military confrontation, high-altitude weapons and ammunition development, and deep-earth protection engineering.

## Introduction

Explosives in the air when the explosion, the instantaneous generation of high-temperature and high-pressure explosion products, and violently compressed around it, forming a layer of compressed interface, that is, the shock wave front. At the same time, the formation of sparse waves inside the blast products, from the blast-air interface to the center of the blast propagation. As the explosion products and shock waves continue to propagate forward, when the blast product reaches the limit of the volume, the blast product will stop expanding, the shock waves can be considered separate from the explosion products.

**Figure 1:**
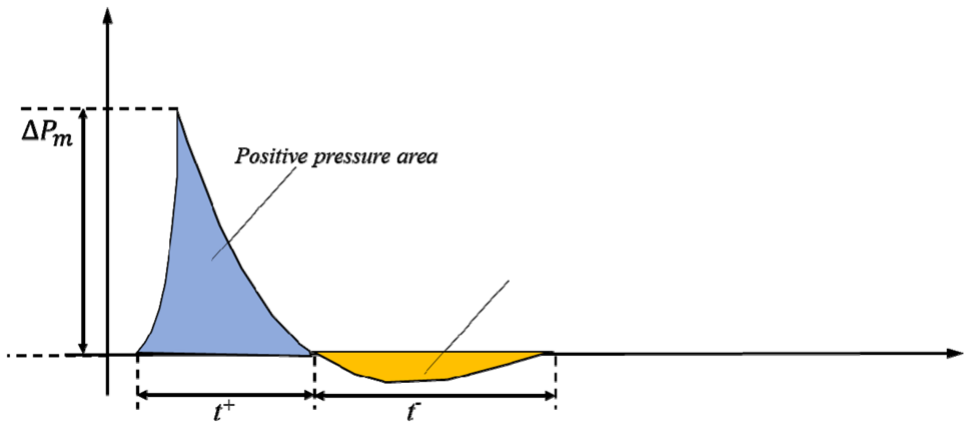
Time history curve of typical shock wave overpressure.

Explosive products impact and compress the air around the explosion source, resulting in a certain distance from the charge center, the shock waves pressure has never disturbed the state of sudden, resulting in a great positive pressure, forming a positive pressure zone. With the increase of propagation time, the air behind the shock waves front begins to expand, resulting in a continuous decline in pressure, which forms a negative pressure zone below the initial pressure. In the process of free propagation of shock waves, the intensity of waves will gradually decay with the increase of propagation distance, and finally decay to sound waves. The pressure–time curve of its propagation process is shown in the following Fig. 1.

Explosives in the free field explosion, high temperature and high pressure gas within an instant. The explosion products will violently spread in all directions and compress the surrounding air, forming shock waves. Compared with the open free space, the propagation state of the shock waves in the confined space is much more complex, multiple reflections are superimposed, and the peak pressure generated is significantly increased. The effect of the shock waves generated by the explosion on various objects is mainly measured by the peak overpressure *∆p*, specific impulse *i* propagation velocity *u*, and other parameters. It is generally considered that the explosive blast damage in the form of shock waves damage. The relationship between peak pressure, specific impulse and shock waves propagation velocity in order to find out, reveal the rules of shock waves propagation and the mechanism of damage, domestic and foreign scholars have conducted a large number of experimental and theoretical studies and put forward a series of semi-empirical formulae for calculating the shock waves overpressure^1–4^.

When the ambient pressure or temperature and other external conditions change, the peak pressure, specific impulse and blast waves propagation speed, and other parameters will also be corresponding changes, especially when the initial ambient pressure changes, the propagation characteristics of the shock waves compared to the atmospheric pressure case will change significantly. Regarding the propagation rules of explosion shock waves under different pressure environments, scholars at home and abroad have carried out different studies, and have achieved some valuable research results. The explosion products in a vacuum environment was studied by Zhang et al.^5^ by experiments, it was concluded that the vacuum environment, the propagation of the explosion products had a significant directional, energy decay is also more rapid. Li et al.^6^ simulated the changes of the characteristic parameters of the explosion near-field of ammonium oil explosives under different degrees of vacuum by using the simulation software AUTO-DYN, and developed an overpressure calculation formula applicable to the explosion near-field of ammonium oil explosives. Using an evacuation device, Zhu^7^ experimentally concluded that the shock waves pressure at the rifling port decreases approximately linearly with the decrease of ambient pressure. You et al.^8^, varying the initial ambient temperature and pressure, conducted experiments on C5–C6 in-tube detonation of hydrocarbon fuels, and the results showed that the effect of temperature on the detonation parameters was much smaller than that of the initial pressure under ambient conditions. By conducting explosion experiments in a negative pressure environment, Wang et al.^9^ showed that the peak vibration velocity of the cylinder and the sound pressure level of the explosion noise tended to decrease with the decrease of the initial pressure, and the main vibration frequency of the cylinder decreased with the decrease of the initial pressure.

Xie et al.^10^ studied the propagation rules of blast shock waves at different altitudes from the characteristics of one-dimensional spherical blast air shock waves. He based on the cloudburst formula proposed by Orlenko, the derivation to the plateau environment, a comparison of the change in shock waves parameters at different altitudes, quantitatively gives the degree of influence of plateau altitude on the propagation characteristics of explosive shock waves. Song et al.^11^ used the finite element software LS-DYNA to simulate the shock wave propagation rules when the center of a confined structure is detonated under different degrees of vacuum, and concludes that when the scale distance is above 0.8, LS-DYNA can only be applied to simulate pressure fields with an initial pressure of 0.01–0.06 MPa. Jack Jr et al.^12^ performed simulations of high air burst to derive shock wave characteristics that do not satisfy Sachs' law. Under different conditions of atmospheric pressure (81.4 kPa, 101.3 kPa, 156.5 kPa), Veldman et al.^13^ conducted experimental and numerical studies on the reflected shock waves pressure and impulse and found that the reflected impulse was more sensitive to changes in ambient pressure as the distance between the charge and the reflected structure increased. Silnikov et al.^14^ studied the effect of initial pressure on the quasistatic component after the blast load, and experimentally demonstrated that the shock waves effect from a blast in an environment below normal atmospheric pressure is less than the effect from a similar blast occurring at normal atmospheric pressure. Izadifard et al.^15^ studied the effect of ambient pressure on various shock waves parameters and showed that the overpressure above sea level height is less than the overpressure at sea level height. Above research on shock waves either in a single pressure environment or numerical simulation of different pressure environments, but the experimental studies on the propagation of explosive waves in confined spaces under different vacuum levels have not been reported, the lack of systematic experimental research in this area, different initial environmental pressure of the explosion theory system has not been formed.

At present, the study of the initial environmental pressure on the rule of explosive shock wave propagation is mainly numerical simulations of software such as AUTODYN together with a limited number of experiments, and most of them are negative pressure experiments. For a larger range of initial pressure, especially multi-gradient negative pressure, positive pressure environment on the impact of explosive shock waves lack of systematic and comprehensive comparative study.

In this paper, Theoretical analysis of the effect of different initial ambient pressures on blast shock wave parameters, the use of small adjustable initial pressure column explosive container to carry out experimental studies of explosive blast shock wave propagation under different initial environmental pressure, to explore the impact of initial pressure on the propagation of explosive shock wave and the rules of change of explosive gas products.

## Theoretical analysis of explosion shock wave parameters

### Dimension analysis of explosion shock wave peak overpressure

The physical quantities that affect the shock wave parameters of explosive explosion in air are : total energy *E* released by explosive explosion, air environmental pressure *p*, air density *ρ* and propagation distance *r*. Ignoring the viscosity and heat conduction of air medium, the peak overpressure of explosion shock wave can be expressed as a function of air parameters :

1 $$ \Delta p_{{\text{m}}} = f_{1} \left( {E0,ph,\rho 0,r,{\text{t}}} \right). $$

From the Π theorem, it can be seen that there are 3 fundamental measures in Eq. (1): *M*, *L* and *T*, corresponding to 3 independent reference physical dimensions, and choosing *E*, *p* and *ρ* as independent variables, the combined measure of the measure is 3.

Let the dimensionless combination of $\lambda$_1_ be:

2 $$ \pi 1 = \frac{r}{{E_{0}^{{^{a1} }} \cdot p_{h}^{a2} \cdot \rho_{0}^{a3} }}. $$

Then:

3 $$ L = (ML^{2} T^{ - 2} )^{{a_{1} }} (ML^{ - 1} T^{ - 2} )^{{a_{2} }} (ML^{ - 3} )^{{a_{3} }} . $$

$$ \begin{aligned} & L: \, 1 = 2a_{1} - a_{2} - 3a_{3} \\ & M: \, 0 = a_{1} + a_{2} + a_{3} \\ & T: \, 0 = - 2a_{1} - 2a_{2} . \\ \end{aligned} $$

Calculated:

4 $$ \pi 1 = \frac{r}{{\sqrt[3]{{{\raise0.7ex\hbox{${E0}$} \!\mathord{\left/ {\vphantom {{E0} {ph}}}\right.\kern-\nulldelimiterspace} \!\lower0.7ex\hbox{${ph}$}}}}}}. $$

Similarly, we can derive:

5 $$ \pi 2 = \frac{t}{{E_{0}^{{^{\frac{1}{3}} }} \cdot p_{h}^{{^{{\frac{ - 5}{6}}} }} \cdot \rho_{0}^{{^{\frac{1}{2}} }} }} = \frac{c0t}{{\sqrt[3]{{{\raise0.7ex\hbox{${E0}$} \!\mathord{\left/ {\vphantom {{E0} {ph}}}\right.\kern-\nulldelimiterspace} \!\lower0.7ex\hbox{${ph}$}}}} \cdot \sqrt k }}, $$

6 $$ \pi 3 = \frac{{\Delta p_{{\text{m}}} }}{{p{\text{h}}}}. $$

*c*_0_ is sound velocity.

Substitute (4), (5) and (6) into (1):

7 $$ \frac{{\Delta p_{{\text{m}}} }}{ph}{ = }f\left( {\frac{r}{{\sqrt[{3}]{{{\raise0.7ex\hbox{${E0}$} \!\mathord{\left/ {\vphantom {{E0} {ph}}}\right.\kern-\nulldelimiterspace} \!\lower0.7ex\hbox{${ph}$}}}}}},\frac{c0t}{{\sqrt[{3}]{{{\raise0.7ex\hbox{${E0}$} \!\mathord{\left/ {\vphantom {{E0} {ph}}}\right.\kern-\nulldelimiterspace} \!\lower0.7ex\hbox{${ph}$}}}} \cdot \sqrt k }}} \right). $$

For the pressure that the shock wave reaches the moment (*t* = *0*) at different distances, the relation of peak pressure of shock wave is as follows:

8 $$ \frac{{\Delta p_{{\text{m}}} }}{ph}{ = }f\left( {\frac{r}{{\sqrt[{3}]{{{\raise0.7ex\hbox{${E0}$} \!\mathord{\left/ {\vphantom {{E0} {ph}}}\right.\kern-\nulldelimiterspace} \!\lower0.7ex\hbox{${ph}$}}}}}}} \right). $$

Under the same conditions, the energy released by explosive explosion is only related to the charge mass *m*_e_

9 $$ \frac{{\Delta p_{{\text{m}}} }}{ph}{ = }f\left( {\frac{r}{{\sqrt[{3}]{{{\raise0.7ex\hbox{${E0}$} \!\mathord{\left/ {\vphantom {{E0} {ph}}}\right.\kern-\nulldelimiterspace} \!\lower0.7ex\hbox{${ph}$}}}}}}} \right) = f\left( {\overline{R} \cdot p_{h}^{{^{\frac{1}{3}} }} } \right), $$

where, $$pv^{n} = p_{H} v_{H} ,p_{K} \le p \le p_{H} \, $$.

It can be seen that the environmental pressure *p*_*h*_ has an effect on the explosion shock wave overpressure Δ*p*_m_.

### Analysis of blast waves peak overpressure variation and velocity

The expansion of detonation products starts from the C–J point. Due to the short time from the C–J pressure formed by explosive explosion to the initial shock wave formed by contact with the medium, it can be approximately regarded as the isentropic expansion process of ideal gas. For the case of explosion products flying into the air, it can be assumed that the action process is one-dimensional, the parameters of the initial interface are considered, and the following two segmented insulation lines are used to replace the expansion adiabatic curve in the actual process^16^.

10 $$ pv^{n} = p_{H} v_{H} ,p_{K} \le p \le p_{H} {,} $$

11 $$ pv^{k} = p_{k} v_{K}^{k} ,p \le p_{K} {.} $$

In the formula, the isentropic exponent *n* and *k* can be 3 and 1.2, respectively. ${p}_{H}$ and ${v}_{H}$ are the parameters of detonation products at the detonation wave front, *D* is the detonation velocity of explosive, ${c}_{K}$ is the particle velocity at *K* point. ${p}_{K}$ and ${v}_{K}$ are the detonation product parameters at point *K*, and their values can be determined by the hugoniot equation of detonation waves.

The following formula can determine the expansion rate of detonation products^16^.

12 $$ u_{x} = u_{H} + \frac{{2c_{H} }}{n - 1}\left( {1 - \frac{{c_{K} }}{{c_{H} }}} \right) + \frac{{2c_{k} }}{k - 1}\left( {1 - \frac{{c_{x} }}{{c_{K} }}} \right). $$

Transformed

13 $$ v_{x} = \frac{D}{n + 1}\left\{ {1 + \frac{2n}{{n - 1}}\left[ {1 - \left( {\frac{{p_{K} }}{{p_{H} }}} \right)^{{\frac{n - 1}{{2n}}}} } \right] + \frac{{2c_{K} }}{k - 1}\left[ {1 - \left( {\frac{{p_{x} }}{{p_{K} }}} \right)^{{\frac{k - 1}{{2k}}}} } \right]} \right\}. $$

The initial pressure of shock waves can be determined by the following equation^16^.

14 $$ p_{x} = \frac{k + 1}{2}\rho_{a} v_{x}^{2} . $$

${p}_{x}$ is the initial pressure of shock wave, *ρ*_*a*_ is the initial air density.

Combining the above two formulas and substituting *n* and *k* to simplify:

15 $$ v_{x} + 10c_{K} \left( {\frac{{1.1\rho_{a} v_{x}^{2} }}{{p_{K} }}} \right)^{\frac{1}{12}} = \frac{D}{4}\left( {2 - \left( {\frac{{p_{K} }}{{p_{H} }}} \right)^{\frac{1}{3}} } \right) + 10c_{K} . $$

According to Izadifard^15^, assuming that the internal energy and temperature of air are constant, the relationship between pressure and density under different vacuum degrees can be simplified as follows:

16 $$ \rho_{1} { = }\frac{{p_{1} }}{{p_{0} }}\rho_{0} , $$

where *ρ*_1_ and *p*_1_ are the air density and pressure at a certain environment; *ρ*_1_ and *p*_0_ are the density and pressure of air at atmospheric pressure, respectively.

According to the analysis of Eqs. (7–10), when the density of air decreases, that is, when the pressure of air decreases, the expansion rate of explosive products increases. The re-observation (14) shows that the higher the initial density of air, the greater the initial shock wave intensity. The initial shock wave velocity can be simplified by the following formula^16^.

17 $$ D_{x} = \frac{k + 1}{2}v_{x} . $$

It can be seen from Eq. (17) that the larger ${v}_{x}$ is, the larger ${D}_{x}$ is, namely, the smaller the initial air density is, the larger the initial velocity of shock wave is. Of course, the analysis process considers that the values of *n* and *k* are constant, and the analysis in this case is ideal. However, the correct conclusion can still be obtained for the qualitative analysis.

## Experimental setup

### Explosive vessel design

**Figure 2:**
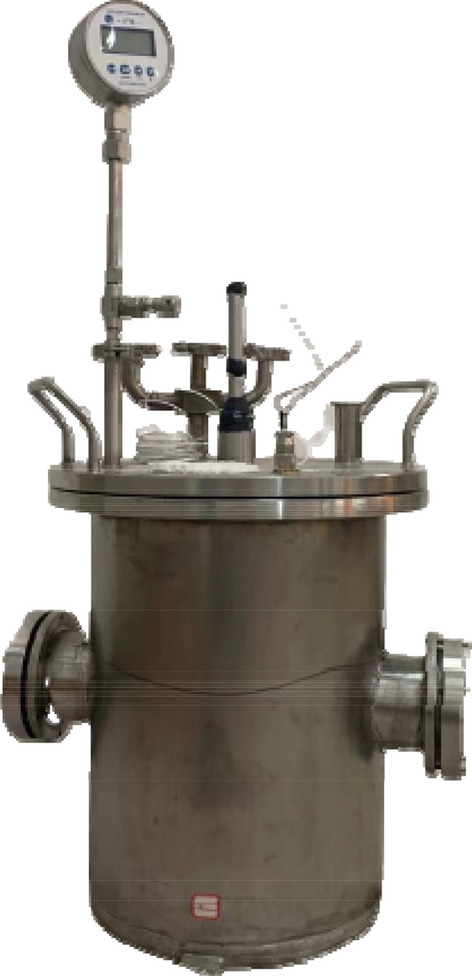
Adjustable initial pressure column explosion vessel.

The developed explosion vessel is shown in Fig. 2. The explosive vessel is a cylinder, and the main material is made of stainless steel, the vessel height is 43.3 cm, inner diameter is 37.5 cm, outer diameter is 38.7 cm, and the wall thickness is 0.6 cm.

### Experimental system

**Figure 3:**
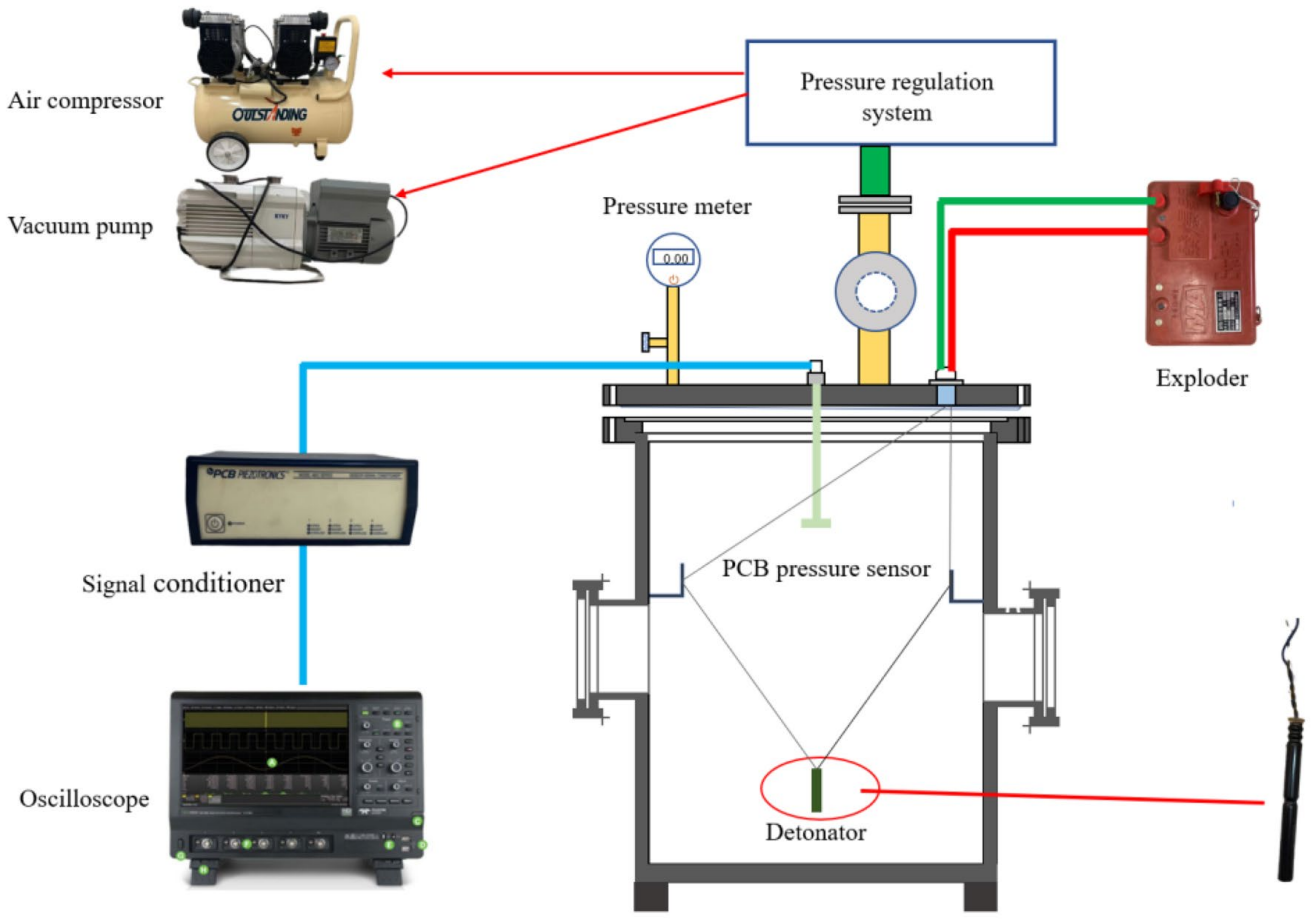
Experimental testing and data collection systems.

The test system consists of an explosion vessel, a digital pressure gauge, a digital vacuum gauge, a PCB pressure transducer (113B24), a signal conditioner, a Lecroy oscilloscope, and a pressure regulation system. The pressure regulation system consists of a vacuum pump and an air compressor. Both the experimental setup and the test system are shown in Fig. 3.

An industrial electric detonator (approximately 1.07 g of TNT equivalent) was used as the source of the explosive, with the concentrating point downward and perpendicular to the bottom of the vessel. The bottom of the detonator was 3.9 cm from the bottom of the vessel. The PCB pressure sensor is fixed on the vessel axis, directly above the explosion source, which is adjustable up and down by means of a thread, and the sensitive surface was perpendicular to the vessel axis. The pressure adjustment system was used to adjust the different pressure environments and the digital pressure gauge to observe the pressure inside the vessel to achieve different initial pressure environments in the explosion vessel. In the same burst center distance point tested explosives in 1.4 atm, 1.2 atm, 1.0 atm, 0.8 atm, 0.6 atm and other different pressure environments in the vessel explosion shock waves reflection overpressure data, to obtain the overpressure time curve.

## Experimental results and discussion

### Analysis of the propagation process of the explosion waves in the explosive vessel

**Figure 4:**
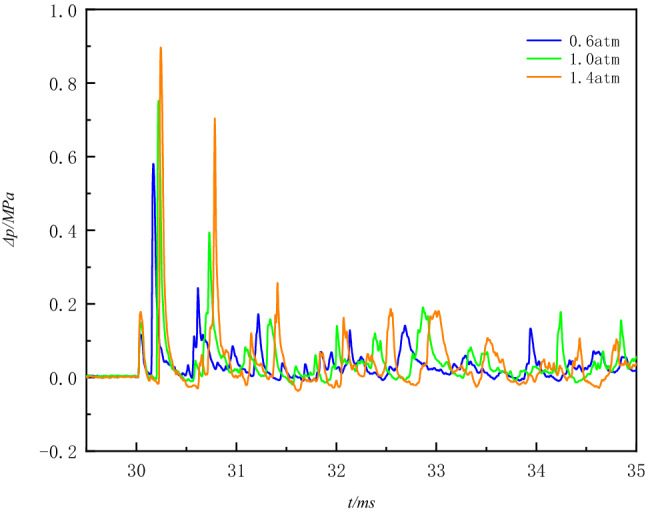
*∆p–t* curves of explosion waves at different ambient pressures.

When explosives explode in the explosive container, the explosion products quickly compress the surrounding gas, resulting in a rapid rise in pressure in a very short period of time to a maximum. As the shock waves continue to propagate forward when the shock waves impact the container wall or the bottom of the container and other places will appear superimposed and converge, depending on the structure of the container, the reflection of the situation is different. When a superimposed reflection of the shock waves occurs, the pressure increases dramatically, up to several times the initial explosion pressure. Depending on the type of reflection, reflected intensity also varies^7^. When the initial pressure inside the vessel is different, the state of spreading also changes, and as the shock waves propagate, a gradual attenuation of energy within the vessel. Figure 4 shows the overpressure time history curve under different pressure conditions.

Compared to the case of explosives exploding in free space, there are some unique features of the overpressure time history curves. In the vessel, shock waves are reflected several times so that the overpressure time history curve has multiple peaks, and the second pressure peak is higher than the first pressure peak. An irregular negative pressure zone is present in the back half of the curve, and occasionally the back peak is higher than the front peak. As the initial pressure increases, the time interval, between the first and second peak pressures, are increasing.

The first pressure peak *p*_1_ is the overpressure of the shock waves acting directly perpendicular to the sensitive surface of the sensor after the explosion without any reflection. Because the experimental setting of the detonator center to the bottom of the container distance L_2_ = 3.9 cm than the vessel radius R = 18.8 cm has a clear distance advantage, the overpressure of the first normal reflection of the blast shock wave through the bottom of the vessel reached the sensor significantly faster than the overpressure of the first oblique reflection through the annular wall of the vessel.

### Variation of peak overpressure

With a fixed detonator height, the blast waves pressure is measured separately for different initial pressure conditions. The calculation of the blast waves overpressure can be obtained by the following equation^17^:

18 $$ \Delta P = \frac{{V_{m} }}{{S_{q} }}. $$

*V*_*m*_ is peak voltage of oscilloscope (V), is sensitivity of pressure sensors (V/MPa), The average value of *S*_*q*_ after two calibrations was 716.55 mV/MPa.

**Figure 5:**
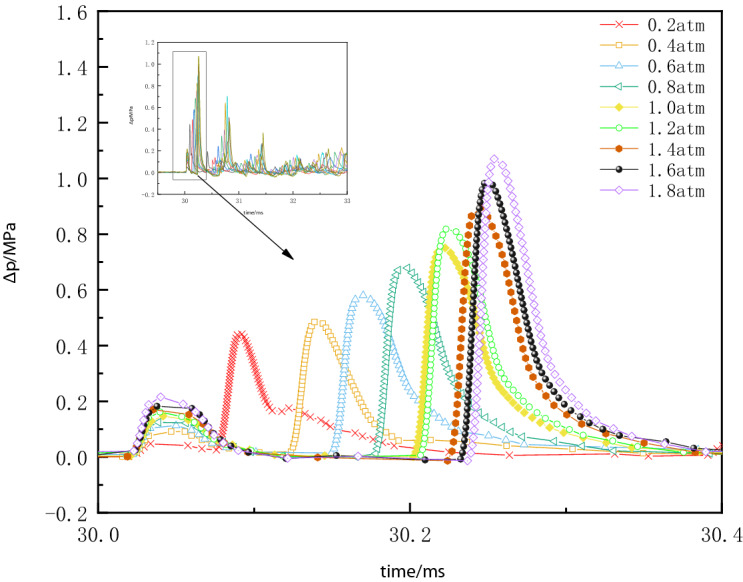
*∆p–t* curves of explosion waves at different ambient pressures.

The time of blast waves propagation to the contact surface of the sensor was set as a uniform moment, and the *Δp–t* curves are measured for different initial pressures in the vessel as shown in Fig. 5. The first overpressure peak and the second overpressure peak of the blast waves with the initial pressure in the vessel are shown in Fig. 5.

**Figure 6:**
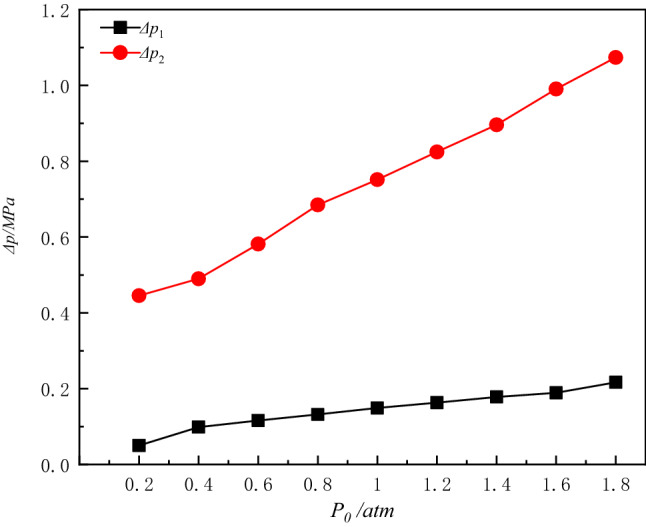
Variation of the first and second overpressure peaks with the initial pressure.

As can be seen from the analysis of Figs. 5 and 6, and the free field explosion shock waves propagation is different, the shock waves inside the vessel due to the constraints of the container wall, there are multiple blast waves reflection superimposed on the situation, there are multiple blast waves pressure peaks. While the second pressure waves peak is the detonator explosion generated by the pressure waves first propagated to the bottom of the vessel, and then produced a reflection superposition. Consequently, a significant rise is shown in the second pressure peak of the shock waves. As the initial pressure increases, the difference in pressure between the first and second peak pressure increases.

**Figure 7:**
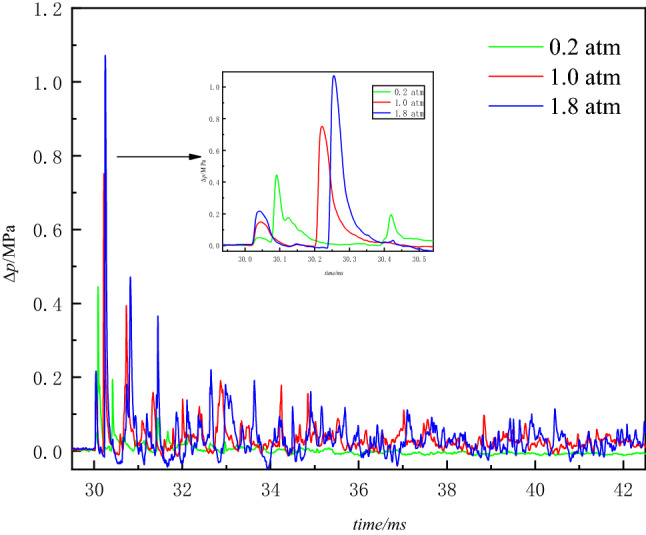
*∆p–t* curves of blast waves at three different ambient pressures.

As the initial pressure inside the vessel increases, the arrival time of the second pressure waves peak is delayed, the initial pressure conditions of the explosion vessel will have a significant impact on the propagation state of the explosion shock waves. In different conditions of the initial pressure, when the blasting center distance is constant, the peak pressure of the explosion waves increases with the initial pressure inside the vessel. As can be seen, reducing the density of the gas medium inside the vessel can effectively reduce the destructive effect of the explosion waves. With the reduction of the initial pressure in the container, the air density compared to atmospheric pressure has been gradually thinner, the explosion generated by the energy propagation depends largely on the explosion products, as can be seen in Fig. 7, with the initial ambient pressure reduction, due to the lack of air medium, the explosion waves energy propagation decay gradually accelerated, it is more difficult to form multiple reflections.

### Velocity variation of the blast waves

**Figure 8:**
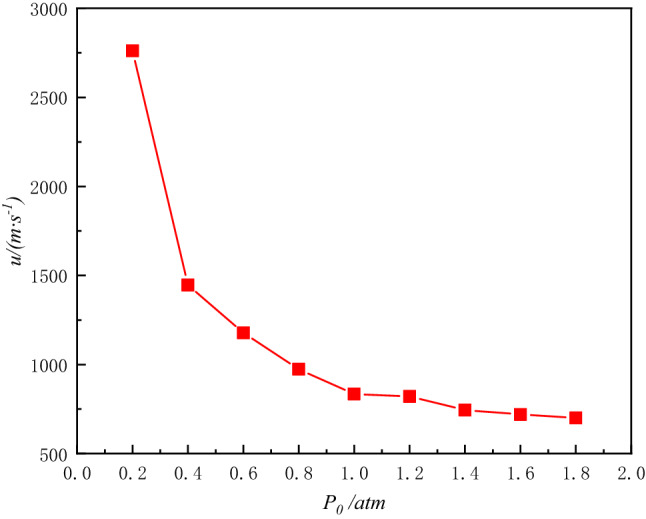
Shock waves velocity under different environmental pressure.

In order to facilitate the analysis of the effect of the initial pressure inside the vessel on the velocity of the blast waves propagation, the velocity of the blast waves is considered constant throughout the process from the blast waves propagation to the pressure sensor. The propagation speed of the blast waves can be obtained by recording the propagation time and distance. The following Table 1 and Fig. 8 show the waves time difference and the blast waves velocity.

**Table 1:** Time difference of waves crest and velocity of explosion waves at different environmental pressures.

Initial pressure/atm	$\Delta t$/*μs*	*u/*(m s^−1^)
1.8	217	700.0
1.6	211	719.9
1.4	204	744.6
1.2	185	821.0
1.0	182	834.6
0.8	156	973.7
0.6	129	1177.4
0.4	105	1446.6
0.2	55	2761.6

As the initial pressure inside the vessel increases, the time for the blast waves to reach the same measurement point gradually increases and its average propagation velocity decreases. It can be seen that the ambient pressure varies in a certain interval, and there is no correlation between the increase in shock waves overpressure and the decrease in waves velocity.

### Specific impulse variation rules

The specific impulse generated by the blast shock waves can be calculated according to the following equation:

19 $$ i = \int_{0}^{t} {\Delta pd\tau } {,} $$

where *t* is the compression duration, *∆p* is the overpressure value, and *τ* is the positive pressure action time.

**Figure 9:**
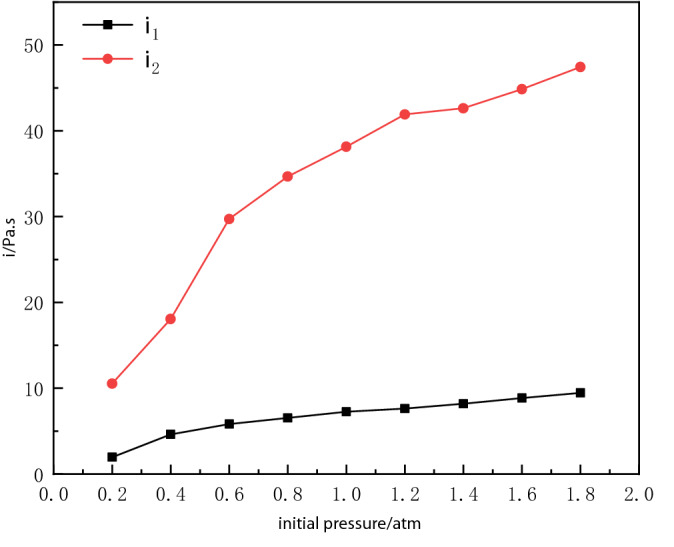
Variation of impulse with initial pressure in vessel.

**Figure 10:**
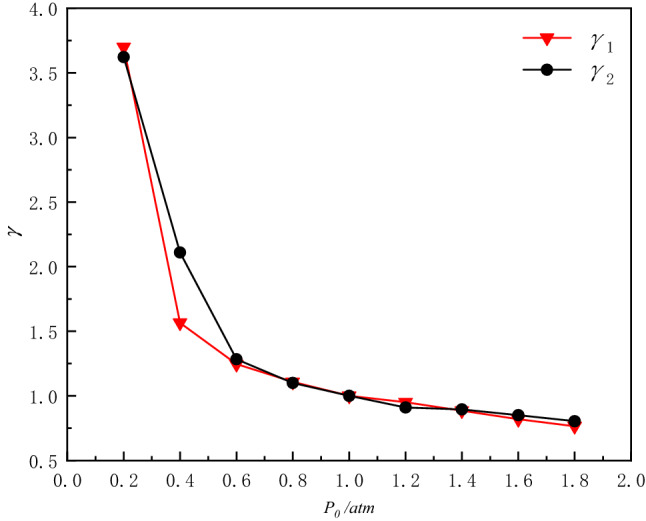
Variation of relative specific impulse factor with initial pressure.

The relative specific impulse factor γ is defined as the ratio between the specific impulse at 1 atm and the specific impulse at different initial values in the explosive vessel. The calculation results are shown in the Figs. 9 and 10.

20 $$ \gamma_{1} \left( {x\,{\text{atm}}} \right) = \frac{{i_{1} \left( {{1}\,{\text{atm}}} \right)}}{{i_{1} \left( {x\,{\text{atm}}} \right)}}{,} $$

21 $$ \gamma_{2} \left( {x\,{\text{atm}}} \right) = \frac{{i_{2} \left( {{1}\,{\text{atm}}} \right)}}{{i_{2} \left( {x\,{\text{atm}}} \right)}}{.} $$

The variation rules of the specific impulse *i*_1_ and the specific impulse *i*_2_ of the 1st blast waves at different initial pressures are given in Fig. 9. As can be seen from Fig. 9, the specific impulse *i*_1_ and *i*_2_ also increase as the initial pressure of the vessel increases. In the range of 0.4 atm to 0.8 atm, the specific impulse *i*_1_ changes more slowly, in the range of 0.2 atm to 0.4 atm, the specific impulse *i*_1_ decreases more rapidly, the specific impulse *i*_2_ decreases more rapidly after 0.6 atm, especially in the range of 0.4 atm to 0.6 atm. In the positive pressure range, the specific impulse *i*_2_ rises more slowly, compared to *i*_1_ which raises the rate significantly (Supplementary Information).

The relative specific impulse factors *γ*_1_ and *γ*_2_ reflect the decay of the first and the second specific impulse of the explosion waves inside the vessel, respectively, and the larger value of *γ* indicates the faster decay. When the initial pressure inside the vessel changes, the highest decay of the first specific impulse *i*_1_ 72.97%, the highest decay of the second specific impulse *i*_2_ 72.39%.

**Figure 11:**
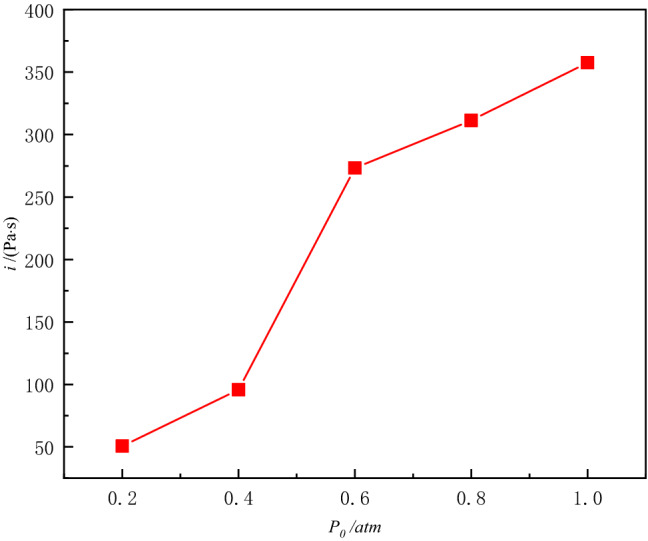
Variation of impulse factor with initial pressure.

From Figs. 9 and 10 can be found in the negative pressure environment than the amount of impulse changes, calculate the negative pressure environment within 10.0 ms after detonation of the specific impulse, resulting in Fig. 11. Analysis can be seen that there is a negative pressure environment with a sharp reduction in overpressure pressure, defined as overpressure-sensitive negative pressure *P*_cr_ this experimental conditions *P*_cr_ is nearly 0.6 atm region within a certain value.

### Effect of gas products on shock waves

The main charge of the detonator used in the experiment is hexogen (RDX), and its explosion equation is as follows.

$$ C_{6} H_{6} N_{6} O_{6} = 3H_{2} O + 3CO + 3N_{2} . $$

The amount of gas produced by the detonator explosion is *n* = 0.0424 mol.

Experiments found that, regardless of the initial negative pressure set in the explosive container, each explosion before and after the stability of the digital pressure difference in the value of about 2.8 kPa, that is, the incremental value of air pressure in the container was about 2.8 kPa. Explosive container volume is 34.8 L, according to the ideal gas equation of state, the increment of gas in the container is calculated as follows:

22 $$ n_{2} = \frac{PV}{{RT}} = 0.039\,{\text{mol}}{.} $$

*n*_*0*_ is the amount of original gas in the explosion vessel, *Δn* is the increase of gas in the vessel after the explosion, and *k* is gas increment percentage.

Theoretical calculations and experiments measured after the explosion of gas generation is relatively close, indicating that the difference in the initial pressure environment does not affect the amount of experimental detonator explosion gas products. As shown in Table 2, the lower the pressure of the initial environment, the amount of explosion-generated gas relative to the amount of gas in the original container percentage of the larger.

**Table 2:** Percentage of gas increment in the vessel.

*P*_*n*_/atm	*n*_*0*_/mol	*Δn*/mol	*k*/%
0.2	0.328	0.039	11.89
0.4	0.636	0.039	6.13
0.6	0.944	0.039	4.13
0.8	1.252	0.039	3.12
1.0	1.56	0.039	2.50
1.2	1.868	0.039	2.09
1.4	2.176	0.039	1.79
1.6	2.484	0.039	1.57
1.8	2.792	0.039	1.40

## Conclusion

In this paper, we designed a ϕ320 mm × 430 mm small adjustable pressure column explosion vessel, conducted different initial pressure explosion test, tested the explosion parameters of industrial detonators at different initial pressures and estimated the explosion shock waves propagation velocity. The main conclusions were obtained as follows.

1. Under the conditions of constant explosion equivalent and distance from the burst center, the overpressure of the shock wave decreases with the initial ambient pressure of the explosion. Explosion shock wave speed size and propagation medium density, the lower the initial environmental pressure, the thinner the gas, the faster the shock wave propagation. The propagation velocity of an explosive wave increases with a decrease in the ambient pressure, and the propagation velocity at a pressure of 1.8 atm is four times less than the velocity at a pressure of 0.2 atm.
2. With the initial environmental pressure changes, the amount of explosive gas products produced does not change. The greater the initial pressure of the environment in which the explosive is located, the less the amount of gas produced by the explosion relative to the proportion of the initial force gas volume in the explosive container, the influence on the propagation of shock waves is also smaller
3. The relative specific impulse factor γ is defined to measure the attenuation of the specific impulse of the first and second blast waves in the vessel. The maximum attenuation of the first specific impulse *i*_1_ is 72.97% and the maximum attenuation of the second specific impulse $i$_2_ is 72.39%.
4. When the initial pressure inside the tank is low to a certain extent, the energy generated by the explosion will decay rapidly. At this time, the energy transmission mainly depends on the explosive products, and the increase of wave velocity is limited by the velocity of the explosive products.

## Supplementary Information

Supplementary Information 1.

Supplementary Information 2.

## Data Availability

All data generated or analysed during this study are included in this published article and its supplementary information files.
